# Isolation of Chitinolytic Bacteria from European Sea Bass Gut Microbiota Fed Diets with Distinct Insect Meals

**DOI:** 10.3390/biology11070964

**Published:** 2022-06-25

**Authors:** Fábio Rangel, Rafaela A. Santos, Marta Monteiro, Ana Sofia Lavrador, Laura Gasco, Francesco Gai, Aires Oliva-Teles, Paula Enes, Cláudia R. Serra

**Affiliations:** 1 Department of Biology, Faculty of Sciences, University of Porto, Rua do Campo Alegre, Ed. FC4, 4169-007 Porto, Portugal; fjorangel@gmail.com (F.R.); rafaela.santos@ciimar.up.pt (R.A.S.); marta.monteiro@ciimar.com (M.M.); id9451@uminho.pt (A.S.L.); aoteles@fc.up.pt (A.O.-T.); 2 CIMAR/CIIMAR Interdisciplinary Centre of Marine and Environmental Research, University of Porto, Terminal de Cruzeiros do Porto de Leixões, Av. General Norton de Matos s/n, 4450-208 Matosinhos, Portugal; 3 Department of Agricultural, Forest and Food Sciences, University of Turin, Largo P. Braccini 2, 10095 Grugliasco, Torino, Italy; laura.gasco@unito.it; 4 Institute of Science of Food Production, National Research Council, Largo P. Braccini 2, 10095 Grugliasco, Torino, Italy; francesco.gai@ispa.cnr.it

**Keywords:** *Hermetia illucens*, exuviae, *Tenebrio molitor*, spore-formers, *Bacillus*, probiotic, aquafeeds, chitinase, *Bacillus licheniformis*

## Abstract

**Simple Summary:**

The ever-growing human population is increasingly demanding more fish. As a response, aquaculture has become the fastest growing industry in its sector. Alternatives to fish meal, an unsustainable commodity used as the main protein source for carnivorous species, are urgently needed in aquafeeds. Recently, in Europe, seven insect species have been approved as potential ingredients for animal feeds, including fish feed. However, chitin, one of the components of an insect’s exoskeleton, is indigestible for several economically valuable fish species, decreasing fish performance upon inclusion. This work aimed to isolate, from the European sea bass gastrointestinal tract, probiotic bacteria capable of producing chitinases to improve the use of diets containing high levels of insect meal. Based on the enhanced adaptability of gut microbial communities and the selective pressure of chitin-enriched diets on fish gut microbiota, bacteria were first isolated from the gastrointestinal tract of European sea bass fed chitin-enriched diets. Isolates were then comprehensively screened in vitro for important traits such as their ability to utilize chitin, gut-survival aptitude, and biosafety-related issues required to be considered eligible as probiotics by the European Food Safety Authority (EFSA).

**Abstract:**

Insect meal (IM), recently authorized for use in aquafeeds, positions itself as a promising commodity for aquafeed inclusion. However, insects are also rich in chitin, a structural polysaccharide present in the exoskeleton, which is not digested by fish, resulting in lower fish performance. Through the application of a dietary pressure, this study aimed to modulate European sea bass gut microbiota towards the enrichment of chitinolytic bacteria to allow the isolation of novel probiotics capable of improving the use of IM-containing diets, overcoming chitin drawbacks. Five isoproteic (44%) and isolipidic (18%) diets were used: a fish meal (FM)-based diet (diet CTR), a chitin-supplemented diet (diet CHIT5), and three diets with either 25% of *Hermetia illucens* and *Tenebrio molitor* larvae meals (HM25 and TM25, respectively) or *H. illucens* exuviae meal (diet HEM25) as partial FM substitutes. After an 8-week feeding trial, the results showed a clear modulatory effect towards spore-forming bacteria by HM25 and HEM25 diets, with the latter being responsible for the majority of the chitinolytic fish isolates (FIs) obtained. Sequential evaluation of the FI hemolytic activity, antibiotic resistance, total chitinolytic activity, sporulation, and survival in gastrointestinal-like conditions identified FI645 and FI658 as the most promising chitinolytic probiotics for in vivo application.

## 1. Introduction

Incremental demand for aquaculture products, coupled with the unsustainable use of fisheries products as feed ingredients, has driven the aquafeeds industry towards the use of sustainable fish meal (FM) alternatives. Insect meal (IM) has shown great potential as a novel protein and lipid source for fish farming [1]. IM nutritional value can vary with the insect species, development stage, or processing method [2]. Overall, IM has a high protein content (30–68% dry matter (DM)), with suitable amino acid profiles [3]. Moreover, lipid content (10–30% DM) can be reduced through defatting processes, and its fatty acid profile can be modulated [4,5,6].

To date, IM inclusion in aquafeeds has been successfully tested in several fish species and using different insect species, to a maximum of 25% inclusion, replacing up to 100% of FM [7,8,9,10,11,12,13]. However, dietary IM inclusion higher than 25% tends to decrease protein and lipid digestibility [14,15,16,17], mainly due to high chitin content [1,18]. Morono et al. [19] supported this claim by demonstrating, in vitro, that crude protein digestion is mostly affected by dietary chitin levels. Moreover, when the insects’ exoskeleton is removed, and with it, the majority of the chitin content, dietary IM inclusions up to 60% can be achieved without a negative impact on fish performance [20].

Chitin is a structural polysaccharide composed of covalent β (1→4)-linkages between N-acetylglucosamine [21]. In the insect exoskeleton, chitin forms a tightly packed matrix by bonding itself with structural molecules, such as proteins and lipids [21]. In the fish gastrointestinal (GI) tract, the disruption of these chitinous exoskeletons into N-acetylglucosamine monomers is mainly attributed to chitinolytic enzymes (chitinase and chitobiase) [6,22,23]. As such, the lack of these enzymes in the fish GI tract, whether due to the inability of fish to produce them or due to an inefficient production by gut microbiota, renders chitin indigestible. This limits its potential use as an energy source and the digestive enzymes’ access to the entrapped proteins or lipids, thus decreasing IM and diets’ digestibility [15,16,17,24].

Microbiota’s role in fish performance and health has become increasingly acknowledged, playing key roles in fish immune and digestive functions [25,26,27]. The fish gut microbiota is highly variable as its establishment is influenced by several host-related characteristics (e.g., genetic information, development state, immunologic response, the physicochemical profile of the different regions of the GI tract, stress) [26], as well as environmental and dietary factors [28]. Identifying and characterizing these microbial communities and understanding their contribution to nutrient digestion are of special importance, as through enzymatic secretion gut microbes can break down otherwise indigestible dietary components such as chitin [29,30,31,32,33]. Moreover, dietary inclusion of specific microorganisms as probiotics can be a strategy to potentiate nutrient digestion and ultimately improve aquaculture production [34,35].

Based on the high adaptability of gut microbial communities, and the already demonstrated European sea bass (ESB) gut microbiota sensitivity to dietary modulation [35,36,37], this study applied a dietary pressure to selectively induce gut microbiota changes towards chitinolytic microorganisms. Using different insect meals and a chitin-enriched diet, we aimed to isolate aerobic spore-forming chitinolytic bacteria. Aerobic spore-formers were targeted due to their advantageous resistance characteristics and well-documented probiotic properties. With the main objective of obtaining potential probiotics able to degrade chitin, we comprehensively screened the isolates concerning important probiotic and biosafety traits, namely the absence of antibiotic resistance and hemolytic ability and gut-survival performance, and aptitude. The successful isolation of bacteria able to digest chitin may allow obtaining a probiotic or a probiotic mix with potential interest for the aquaculture industry, capable of improving the use of IM-containing diets.

## 2. Materials and Methods

### 2.1. Animals and Experimental Conditions

European sea bass (*Dicentrarchus labrax*) juveniles were acquired from Sonríonansa (Cantabria, Spain) and transported to the Marine Zoology Station (Porto University, Porto, Portugal). Following their arrival, the fish were submitted to a 4-week quarantine period and fed with a commercial diet (18% lipids and 44% protein, Aquasoja Sustainable Feed, Sorgal, Ovar, Portugal). After the quarantine, the fish were divided into 15 groups of 15 fish with an initial mean body weight of 53.7 ± 2.7 g, and the experimental diets were arbitrarily allocated to triplicate groups. The fish were hand-fed to apparent visual satiation twice daily, 6 days per week. The trial lasted 8 weeks and was performed in a recirculating aquaculture system composed of 15 fiberglass tanks of 100 L capacity, thermo-regulated to 22.7 ± 0.3 °C and supplied with a continuous flow of seawater (36.0 ± 0.5 g L^−1^ salinity, circa 7 mg L^−1^ oxygen), with an artificial illumination photoperiod set to 12:12 h light:dark.

### 2.2. Experimental Diets

Five isoproteic (44%) and isolipidic (18%) diets were formulated: a control FM-based diet without IM inclusion (diet CTR); three diets with 25% inclusion of IM, namely partially defatted black soldier fly (*Hermetia illucens*) larvae meal (diet HM25), black soldier fly exuviae meal (remains of the chitinous husk left behind during insect molts) (diet HEM25), or 25% partially defatted yellow mealworm (*Tenebrio molitor*) larvae meal (diet TM25); and a diet similar to the CTR but including 5% commercial chitin (practical grade powder, Sigma, Química, S.L., Sintra, Portugal) (diet CHIT5). The IM composition was as follows on a DM basis: HM (protein: 60.3%; lipid: 6.4%; chitin 6.8%), TM (protein: 69.5%; lipid: 14.1%; chitin 5.2%), and HEM meal (protein: 64.3%; lipid: 8.1%; chitin 7.2%). HM and TM are the most used IMs to replace FM in aquafeeds, while HEM was chosen to evaluate the potential of this insect production residue in gut microbiota modulation. These dietary ingredients were included at 25% as previous reports have demonstrated that 25–30% inclusion intervals have promoted more pronounced effects on the gut microbiota. All dietary ingredients were finely ground, well mixed, and dry pelleted in a laboratory pellet mill (California Pellet Mill, CPM Crawfordsville, IN, USA) through a 2 mm die. The resulting pellets were dried in an oven at 40 °C for 24 h and stored in airtight bags at −20 °C until used. The ingredients and proximate composition of the experimental diets are detailed in Supplementary Table S1 [38]. The DM, protein, lipid, and ash analysis for all the ingredients and experimental diets were carried out according to the Association of Official Analytical Chemists methods [39]. The chitin contents of HM, TM, HEM, and the experimental diets were analyzed according to Guerreiro et al. [40].

### 2.3. Sampling

To guarantee that fish guts were full, fish were fed 4 h before sampling. Two fish were randomly selected per tank and sacrificed with an anesthetic overdose (3.0 mL^−1^ ethylene glycol monophenyl ether). The whole gut (without pyloric caeca) was aseptically excised and carefully squeezed to collect the digesta content. To overcome inter-fish variations, digests were pooled into one sample per tank.

### 2.4. Isolation of Spore-Forming Bacteria from the European Sea Bass Gut Microbiota

Digesta aliquots containing the pooled material were diluted 1:5 (wt:vol) in Bott & Wilson (B&W) salts (1.24% K_2_HPO_4_, 0.76% H_2_PO_4_, 0.1% trisodium citrate, 0.6% [NH_4_]_2_SO_4_, pH 6.7) and resuspended by vortexing thoroughly. Serial dilutions (10^−0^, 10^−1^, 10^−2^, 10^−3^, 10^−4^) of the homogenized samples were heat-treated at 65 °C for 20 min [41] and spread on the surface of Luria Bertani (LB) agar plates to select aerobic spore-forming bacterial isolates. Samples were then incubated for up to 96 h at room temperature (25 ± 1 °C), and colonies obtained from each experimental group were selected based on their different morphologies. All selected isolates were purified by sequential re-streaking on LB agar plates until pure colonies were obtained. Colonies were then numbered and stored at −80 °C in 25% glycerol. All isolates were tested for catalase activity, through colony resuspension in a 3% hydrogen peroxide solution (Sigma, Química, S.L., Sintra, Portugal), and for spore production ability, induced by nutrient exhaustion on Difco Sporulation Medium (DSM) (Becton, Dickinson and Company, Franklin Lakes, New Jersey, USA). Sporulation was confirmed by phase-contrast microscopy [35,41].

### 2.5. Colloidal Chitin Preparation

Colloidal chitin preparation was performed according to [42], with minor modifications. Briefly, powdered chitin from shrimp shells (practical grade powder, Sigma-Aldrich Química, S.L., Sintra, Portugal) was homogenized in concentrated HCl (40 g/600 mL, *w*/*v*) with vigorous stirring for 60 min at 25 °C. The colloidal chitin suspension was then precipitated by slowly adding 2 L of deionized water at 4 °C. Colloidal chitin was sedimented by centrifugation (15,900× *g*, 10 min at 4 °C) and resuspended in 3 L of deionized water. The process was repeated until the suspension pH was 3.5, and then the colloidal chitin was freeze-dried and stored until further use.

### 2.6. Selection of Isolates with Chitinolytic Activity

To select bacteria capable of hydrolyzing chitin, each isolate was grown on a chemically defined medium, hereafter designated ChitMedia (*w*/*v*, 0.65% Na_2_HPO_4_, 0.3% KH_2_PO_4_, 0.05% NaCl, 1% NH_4_Cl, 0.02% MgSO_4_, 0.01% yeast extract, 1% colloidal chitin), containing 1.6% (*w*/*v*) agar, hereafter referred as ChitAgar. Isolates were grown up to 14 days at 28 °C and selected based on their chitinolytic ability, identified by the presence of a hydrolysis halo. *Chromobacterium violaceum* ATCC 12472, known to possess chitinolytic activity, was used as a positive control [43]. 

The chitinolytic activity was determined indirectly, by measuring the N-acetylglucosamine produced by the bacterial culture supernatant. To do so, each isolate was grown in ChitMedia for 6 days at 37 °C at 150 rpm. Then, 2 mL of each culture was centrifuged at 4 °C for 10 min at 13,600× *g*, and the supernatant was collected. A reaction mixture composed of 250 µL of the supernatant, 250 µL of citrate-phosphate buffer (0.15 M citric acid, 0.3 M sodium phosphate dibasic, pH 7), and 250 µL of chitin suspension (5 mg mL^−1^), was incubated for 2 h at 37 °C in a rocking shaker. A sample blank (250 μL supernatant + 500 μL distilled water), a substrate blank (250 μL citrate-phosphate buffer + 250 μL chitin suspension + 250 μL distilled water), and a reagent blank (250 μL citrate-phosphate buffer + 500 μL distilled water) were also prepared. Duplicates were carried out for each sample and blank. After incubation, the reaction was stopped by heating at 100 °C for 10 min and buffered with 0.1 mL of 0.8 M borate buffer (K_2_B_4_O_7_) at pH 9.3, with the remaining protocol being carried out as described by Guerreiro et al. [16]. N-acetylglucosamine concentration was determined by measuring the sample’s absorbance at 585 nm. An N-acetylglucosamine standard curve was carried out (65.5–1000 μM). Enzymatic activity was conveyed in μg of N-acetylglucosamine mL^−1^ of culture supernatant min^−1^. All readings were carried out at room temperature in a Multiskan GO microplate reader (Model 5111 9200; Thermo Scientific, Nanjing, China). All reagents used to perform the enzymatic analysis were purchased from Sigma-Aldrich (Química, S.L., Sintra, Portugal).

The fish isolates (FIs) presenting the highest chitinolytic activity were tested under conditions that mimic the ESB stomach or intestinal pH. For that purpose, the citrate-phosphate buffer was adjusted to pH 5 or pH 7, respectively [44]. The reaction mixtures were then incubated for 2 h in a rocking shaker at 24 °C, which is within the optimum growth temperature for ESB [45]. Following enzyme inactivation at 100 °C for 10 min, to maintain a final reaction pH of 9.8 in the solution, the reaction mixtures were buffered with 0.1 mL of 0.8 M borate buffer (K_2_B_4_O_7_), at either pH 10.8 or pH 9.3, respectively, for the reactions carried out at pH 5 or pH 7. The remaining process was carried out as described above.

### 2.7. Taxonomic Identification of Isolates with Extracellular Chitinolytic Activity

The chromosomal DNA of each isolate was purified from a 2 mL overnight culture grown in LB at 37 °C, incubated in a shaker at 120 rpm, using the ZymoBIOMICS DNA Miniprep Kit (Zymo Research Corp., Irvine, CA, USA), according to the manufacturer instructions. PCR amplification of the 16S rRNA gene was carried out using the 27F and 1492R oligonucleotide primers [46], at an annealing temperature of 55 °C. Each 25 μL reaction was composed of 1 × x MyTaq Reaction Buffer (Bioline, BIOPORTUGAL, Lda., Lisboa, Portugal), 0.2 μM of each primer (STAB Vida, Lisboa, Portugal), 1.25 U of MyTaq DNA Polymerase (Bioline, BIOPORTUGAL, Lda., Lisboa, Portugal), and 2.5 µL of DNA template. Sequencing of the amplified products was carried out using the primer 27F [46] by STABVIDA (Caparica, Portugal). The sequencing data were analyzed by BLASTn against a database of non-redundant bacterial sequences from NCBI (https://www.ncbi.nlm.nih.gov (accessed on 3 May 2020)).

### 2.8. Hemolytic Activity and Antibiotic Resistance

Hemolytic activity was determined using freshly grown LB colonies cultured on Columbia Agar with 5% sheep blood (Becton Dickinson GmbH) at 37 °C for 24, 48, and 72 h. The isolates were identified as β-hemolytic if the bacterial hemolytic enzymes completely broke down the blood cells, α-hemolytic if the bacterial hemolytic enzymes only partially broke down the blood cells, or γ-hemolytic if no hemolytic activity was detected. 

Antibiotic susceptibility testing was carried out by determining minimal inhibitory concentrations (MICs) using Etest (bioMérieux, Inc., Marcy-l’Étoile, France). Following the recommendations of the European Food Safety Authority Panel on Additives and Products or Substances used in Animal Feed (EFSA-FEEDAP) [47], the isolates were tested against 7 antibiotics belonging to 4 different antibiotic classes: erythromycin (E), which belongs to the macrolide class; kanamycin (K), streptomycin (S), and gentamycin (G), which belong to the aminoglycosides class; tetracycline (T), which belong to the tetracycline class; and vancomycin (V) and chloramphenicol (C), which belong to the glycopeptide class. Overnight-grown bacteria were adjusted to an optical density of 0.5 McFarland standard units (OD600 ~ 0.1) in sterile saline solution and homogeneously spread in 20 mL MH (Mueller-Hinton, Oxoid, Fisher Scientific, Oeiras, Portugal) agar plates. Two Etest strips were used per plate. The MIC was determined after 24 h incubation at 37 °C.

### 2.9. Screening for Putative chiA

To tentatively confirm the presence of the chitinolytic domain ChiA, the FIs were screened for the presence of chitinase A-encoding gene (*chiA*) using two sets of primers. Based on the already demonstrated ability to amplify *chiA* from other *Bacillus* species, the first set of primers was used to target a conserved region of ChiA as designed by Ramaiah et al. [48]. Based on the taxonomic proximity of our FIs to *Bacillus licheniformis*, a second set of primers was used. For that purpose, multiple *chiA* sequences belonging to *B. licheniformis* and identified with the accession numbers GQ899144, FJ465148, KJ017976, CP042252, CP041154, and CP023729 were retrieved from the NCBI database (https://blast.ncbi.nlm.nih.gov (accessed on 18 November 2020)) and aligned (Supplementary Figure S1) using ClustalW algorithm with Molecular Evolutionary Genetics Analysis (MEGA) software version 10.2.6 [49]. Conserved regions of aligned sequences were chosen for primer design using Primer3 software [50]. Primer sequences and amplicon size can be found in Supplementary Table S2. PCR amplification was performed as described for the 16S rRNA gene (Section 2.7), with the annealing temperature of 55 °C and 54 °C for the first and second sets of primers, respectively.

### 2.10. Spore Concentration, Sporulation Efficiency, and Spore Viability after Gut Environment Simulation

Sporulation induction was carried out by nutrient exhaustion in DSM for 48 h at 37 °C in an orbital shaker at 150 rpm, with sporulation efficiency being determined as described by [35]. The remainder of the media containing each isolate culture was collected and submitted to heat treatment at 80 °C for 20 min to eliminate vegetative cells, and the cells were harvested by centrifugation at 13,600× *g* for 10 min. Evaluation of the FI resistance to gastrointestinal tract transit was performed by sequentially exposing the heat-resistant cells of the FIs to stomach-like and intestine-like conditions as described by [35]. Determination of the colony-forming units (CFU mL^−1^) was carried out at each evaluation point. Triplicates were used for each isolate. All reagents used were purchased from Sigma-Aldrich (Química, S.L., Sintra, Portugal).

### 2.11. Statistical Analysis

All data were checked for normality and homogeneity of variances before analysis and transformed if necessary. The isolates’ total chitinolytic activity at pH 7 and 37 °C was analyzed by one-way analysis of variance (ANOVA). The isolates’ total chitinolytic activity in gut-like conditions was analyzed by two-way ANOVA with the isolates and pH as the main factors. To identify differences between means, Tukey multiple range tests were performed. Differences were considered statistically significant at *p* < 0.05. All statistical analyses were performed using the software IBM SPSS 27.0 software package for Windows (IBM SPSS Statistics).

## 3. Results

### 3.1. Isolation, Identification, and Characterization of Putative Probiotics with Chitinolytic Potential

A total of 366 heat-resistant colonies with different morphologies were isolated. Of these, 310 isolates were obtained from fish fed the HM-based diets, namely 107 isolates (29.2% of the total) from the HM25 group and 203 isolates (55.5% of the total) from the HEM25 group. The remaining isolates were obtained from CTR, TM25, and CHIT5 diets: 18 (4.9% of the total), 23 (6.3% of the total), and 15 (4.1% of the total) isolates, respectively. 

**Figure 1:**
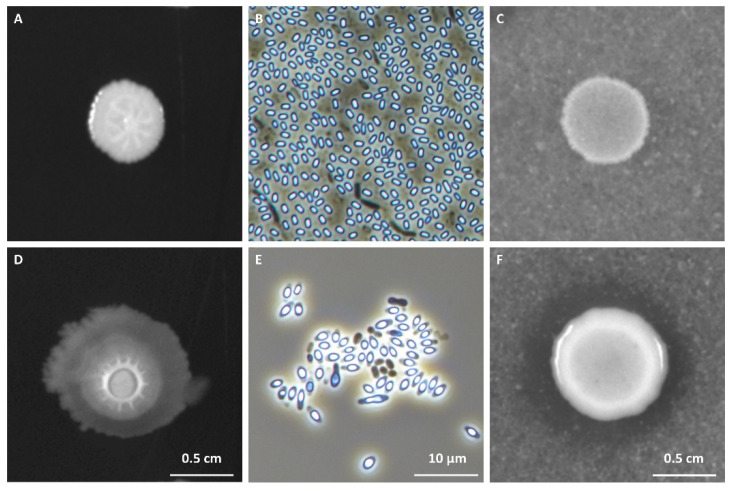
Morphological diversity (Panels (**A**,**D**) of colonies of representative spore-forming fish isolates obtained from European sea bass intestinal contents after a 96 h at 25 °C growth in Luria Bertani (LB) media. Panels (**B**,**E**) depict phase-contrast microscopy images of sporulating cells and free spores of the same 2 representative isolates. Sporulation was induced by nutrient exhaustion in solid Difco Sporulation Medium (DSM). Panels (**C**,**F**) illustrate the criteria for the determination of chitinolytic ability of the isolates in ChitAgar media, with (**E**) representing a negative result (no chitin-degradation halo) and (**F**) representing a positive result (visible chitin-degradation halo).

Following colony purification (Figure 1A,D), sporulation was induced by nutrient exhaustion in DSM, and spore morphology was characterized by phase-contrast microscopy [41] (Figure 1B,E). Results showed that 353 of the 366 isolates (96.4% of total isolates) produced spores. Of these, 346 isolates (98.0% of total isolates) were positive for the catalase testing and were subsequently inoculated into ChitAgar for chitinolytic activity screening. Colonies presenting a hydrolysis halo (Figure 1C,F) were selected, resulting in 27 spore-forming isolates with chitinolytic potential (7.6% of the catalase-positive isolates). These chitin-degrading microorganisms were mainly found in samples from fish fed the HM-based diets (23 out of 27, corresponding to 85.2% of the catalase-positive isolates), with HEM25 having the highest contribution (20 out of 27, corresponding to 74.1%) (Table 1). No spore-forming chitinolytic isolates were identified from fish fed the TM25 diet. Taxonomic identification of the 27 chitinolytic spore-forming isolates by partially sequencing the 16S rRNA gene revealed a predominance of *Bacillus* species (23 out of 27, corresponding to 85.2% of the total), followed by *Paenibacillus* species (4 out of 27, corresponding to 14.8% of the total) (Table 1). The majority of isolates (13 out of 27, corresponding to 48.1% of the total) were closely related to *B. licheniformis*, with the remaining isolates being closely related to *B. tropicus* (1), *B. cereus* (3), *B. paranthracis* (2), *B. clausii* (1), *Bacillus* spp. (3), *P. xylanilyticus* (1), *P. tundrae* (1), *P. dendritiformis* (1), and *P. lautus* (1) (Table 1).

### 3.2. Hemolytic Activity and Antibiotic Resistance

According to EFSA-FEEDAP recommendations, the 27 isolates selected as putative chitinolytic probiotics were tested for minimal biosafety requirements, namely for hemolytic activity and antibiotic resistance. Most isolates did not present hemolytic activity when cultivated on 5% sheep blood agar, thus being classified as γ-hemolytic (20 out of 27, corresponding to 74.1% of the total). The seven isolates presenting β-hemolysis activity were excluded from further analysis. These corresponded to FI568 (closely related to *B. tropicus*); FI633, FI635, and FI670 (closely related to *B. cereus*); FI652 (closely related to *B. clausii*); and FI640 and FI805 (closely related to *B. paranthracis*) (Table 1). The 20 isolates with no hemolytic activity were tested for antimicrobial resistance to seven antibiotics for *Bacillus* and other Gram-positive bacteria (to test isolates closely related to *Paenibacillus*) recommended by the EFSA Panel on Additives Products or Substances used in Animal Feed [47]. Of the 20 isolates, only FI832 presented no antibiotic resistance. Fifteen isolates, closely related to the *Bacillus* genus, were resistant to chloramphenicol, with 12 also being resistant to erythromycin (Table 1). Four isolates, namely FI620, FI622, FI698, and FI710, all closely related to the genus *Paenibacillus*, were resistant to two or more of the following antibiotics: kanamycin, streptomycin, chloramphenicol, tetracycline, vancomycin, and erythromycin (Table 1). Of the isolates tested for antimicrobial resistance, 80% presented resistance against two or more antibiotics, and 15% presented resistance against three or more antibiotics. Erythromycin (65%) and chloramphenicol (85%) were the antibiotics against which most of the isolates presented resistance. The minimum inhibitory concentrations are presented in Supplementary Table S3. Due to the multiple resistances detected, as well as the antibiotic dissemination ability determined by Eduardo-Correia et al. [51], all *Paenibacillus* sp. isolates were excluded from further testing. As such, we proceeded with a strict group of 16 isolates, all belonging to the genus *Bacillus*. 

### 3.3. Chitinolytic Activity of Gut Spore-Formers

**Figure 2:**
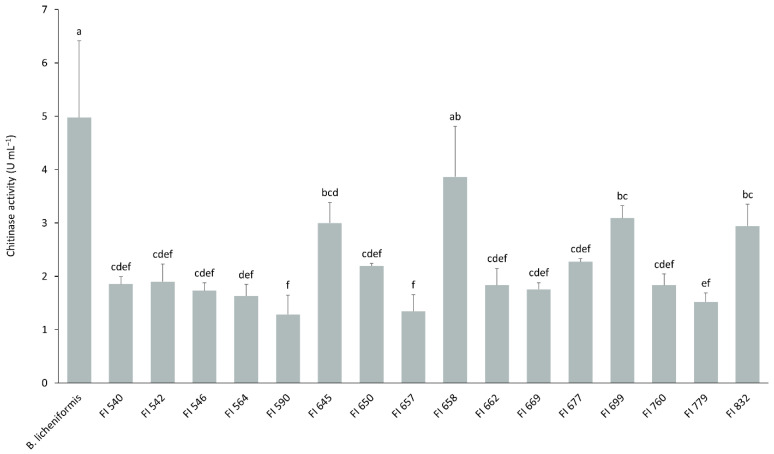
Chitinolytic profile of the selected 16 fish isolates (FI codes in the x axis) obtained from the intestines of European sea bass. Total chitinolytic activity was determined at pH 7 and 37 °C after culturing for 6 days in ChitAgar at 37 °C and 150 rpm. 1U corresponds to the production of 1 µg min^−1^ of N-acetylglucosamine. *B. licheniformis* BGSC 9945A was used as control. The results are the average of three independent experiments and were analyzed by a one-way analysis of variance (ANOVA), followed by a Tukey multiple range test. Error bars represent the standard deviation. Means with different letters indicate significant differences among means (*p* < 0.05).

The 16 isolates selected above were tested for their ability to degrade chitin into N-acetylglucosamine at 37 °C and pH 7. All but one isolate, FI658, presented significantly lower activities than the control (Figure 2). Additionally, isolates FI645, FI699, and FI832 presented a similar chitinolytic activity to that of isolate FI658 and were also selected for further study.

**Figure 3:**
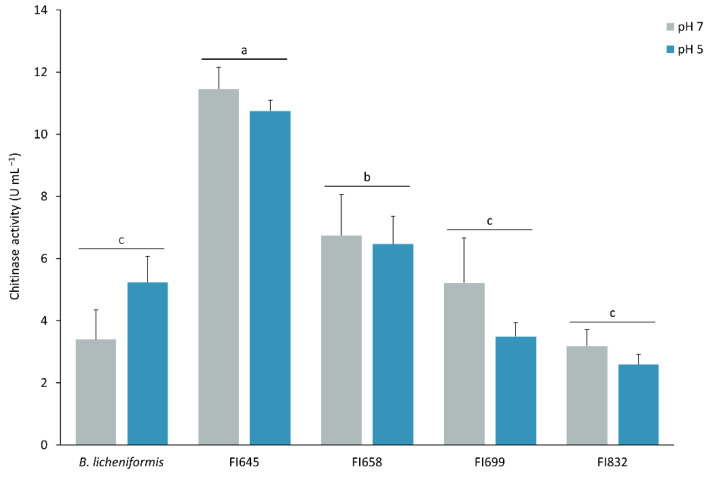
Chitinolytic profile of the best 4 fish isolates (FI codes in the x axis) obtained from the intestines of European sea bass. Total chitinolytic activity of the FIs was determined at pH 5 (blue) and pH 7 (grey) at 24 °C after bacterial culturing for 6 days in ChitAgar at 37 °C and 150 rpm. 1U corresponds to the production of 1 µg min^−1^ of N-acetylglucosamine. *B. licheniformis* BGSC 9945A was used as control. The results are the average of three independent experiments and were analyzed by a two-way ANOVA, followed by a Tukey multiple range test. No differences were found regarding the two pH conditions tested. Independently of the pH, differences between the FIs means are represented by lowercase letters (*p* < 0.05). Error bars represent the standard deviation.

These isolates were further evaluated regarding their in vitro potential to degrade chitin into N-acetylglucosamine at 24 °C and at pH 5 and 7, to mimic ESB stomach and intestine conditions, respectively (Figure 3). In identical growth and enzymatic activity assay conditions, at pH 7, a temperature decrease from 37 °C to 24 °C led to an increased chitinolytic activity of isolates FI645 (3.82-fold), FI658 (1.76-fold), and FI699 (1.69-fold), while it remained unchanged in isolate FI832 (1.09-fold) and decreased in *B. licheniformis* (0.68-fold) (Figure 2 and Figure 3). On the other hand, pH variation at 24 °C had no significant impact on the chitinolytic activity of the isolates (Figure 3). Overall, under gut-like conditions, FI645 presented the highest chitinolytic activity, followed by FI658, with *B. licheniformis*, FI699, and FI832 presenting similar results.

**Figure 4:**
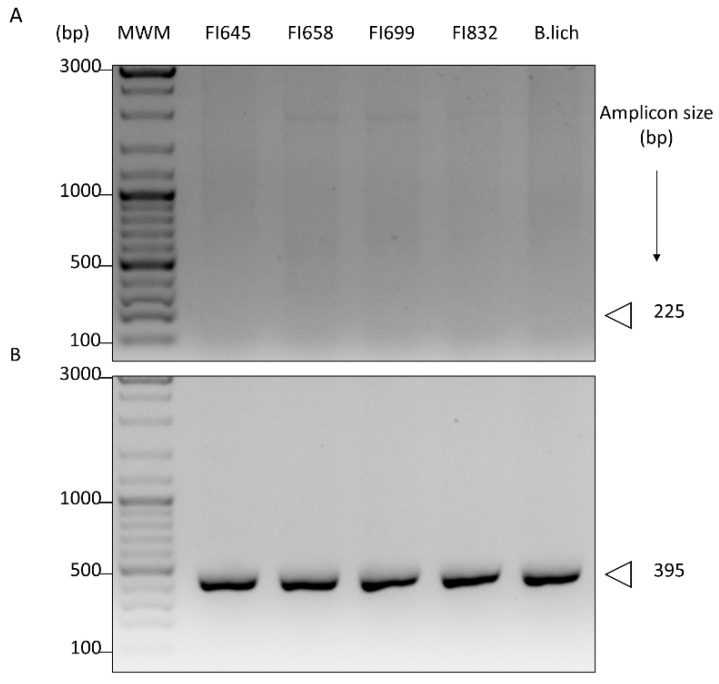
PCR detection of genes coding for the chitinase ChiA protein domain in the genome of fish isolates (FI codes on top of the figure) using (**A**) oligonucleotide primers targeting a conserved region of ChiA, previously designed by Ramaiah et al. (2000), or (**B**) primers targeting conserved regions of *B. licheniformis chiA* designed in this study. Genomic DNA of *B. licheniformis* BGSC 9945A (B. lich) was used as control. The amplicon size, in base pairs (bp), is depicted on the right.

Isolates were also tested for the presence of *chiA*, a gene coding for the catalytic ChiA domain of bacterial chitinases. For this purpose, we first used a pair of oligonucleotide primers designed by Ramaiah et al. [48], which were previously demonstrated to detect *chiA* in a wide array of phylogenetically distant microorganisms, including *Bacillus*. As these primers were not able to detect *chiA* presence in our isolates (Figure 4A), a new set of primers was designed using known *chiA* sequences of closely related microorganisms, namely *B. licheniformis* (Supplementary Table S2 and Supplementary Figure S1). With this new set of primers, a DNA fragment of the expected size was obtained, thus demonstrating the presence of the *chiA* gene in our isolates (Figure 4B). Sequencing of the corresponding DNA fragments revealed an identity >98.9% with previously described chitinase sequences of *B. licheniformis* (data not shown).

### 3.4. Sporulation Efficiency and Resistance to the Gut Environment Simulation

**Figure 5:**
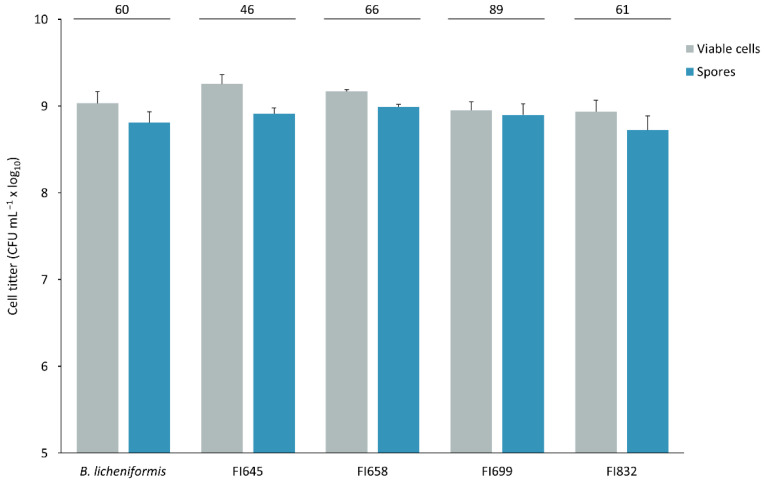
Titer of viable cells of each fish isolate (FI codes in x axis) present in 48 h DSM cultures before (grey, total cells) and after (blue, sporulating or heat resistant cells) a 20 min heat treatment at 80 °C. Sporulation was produced by nutrient exhaustion in liquid Difco Sporulation Medium (DSM) at 37 °C and 150 rpm. The numbers displayed on top of the panel represent the percentages (%) of sporulation calculated as the ratio between sporulating cells and total cells. *B. licheniformis* BGSC 9945A was used as control. The results are the average of three independent experiments. The error bars represent the standard deviation.

**Figure 6:**
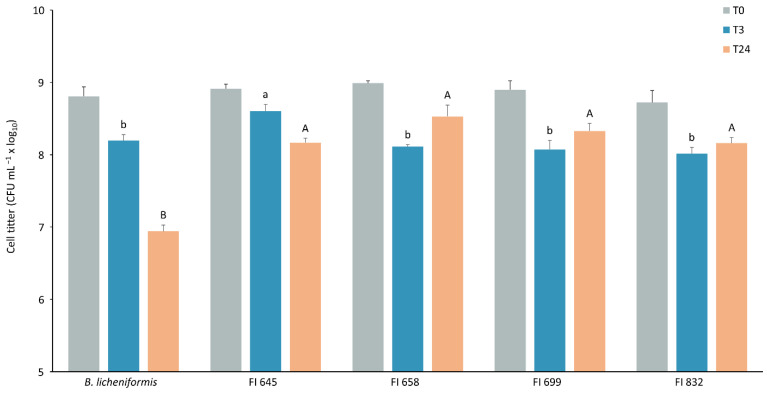
Viability of spores from each fish isolate (FI codes in x axis) before (T0, grey) and after exposure to simulated stomach conditions for 3 h (T3, blue) followed by simulated intestinal conditions for 24 h (T24, orange). *B. licheniformis* BGSC 9945A was used as control. The results are the average of three independent experiments. Data were analyzed by a one-way analysis of variance (ANOVA), followed by a Tukey multiple range test. Error bars represent the standard deviation. Differences in spore viability between strains at T3 are represented by lowercase letters (a/b), while uppercase letters (A/B) represent differences between strains at T24. Statistical significance was considered at *p* < 0.05.

Isolates FI645, FI658, FI699, and FI832 were characterized for their sporulation efficiency and potential to survive passage through the gastrointestinal tract, to establish their suitability for industrial production and feed incorporation. After a 48 h sporulation induction by nutrient exhaustion in liquid DSM medium, all isolates yielded more than 10^8^ heat-resistant cells per milliliter (Figure 5), corresponding to sporulation efficiency values higher than 45%. These spores were then subjected to stomach-like conditions followed by intestine-like conditions. After 3 h in simulated stomach conditions, all isolates maintained over 10^8^ CFU mL^−1^ viable spores, with FI645 presenting the highest spore count when compared to the other isolates and the control. When exposed to intestine-like conditions, the control *B. licheniformis* spore count decreased from 10^8^ mL^−1^ to 10^7^ mL^−1^, being significantly lower than that of all isolates, which maintained a titer > 10^8^ CFU mL^−1^ (Figure 6).

## 4. Discussion

The microbiota’s role in fish performance and health is nowadays well established [25,26,27]. While imbalances in the gut microbiota community can lead to detrimental effects on the host, probiotic administration can enhance fish health and growth performance by promoting a balanced microbiota [27]. To date, several species such as *Bacillus* spp., *Lactobacillus* spp., *Enterococcus* spp., *Streptomyces* spp., and *Carnobacterium* spp. have been successfully used as probiotics in aquaculture [52]. Among these, spore-forming microorganisms such as *Bacillus* spp. are particularly attractive. Their robustness and survival ability in extreme conditions represent a clear advantage for industrial applications, namely for inclusion in animal feeds and for survival inside the animal gut [53,54,55,56].

In this study, dietary inclusion of different IMs and commercial chitin prompted differential shifts in the digesta-associated culturable spore-forming community, with HM25 and HEM25 diets aggregating most of the FIs obtained. The accentuated increase in Firmicutes detected in HM25 and HEM25 diets is in line with our previous work, with both diets showing, nonetheless, different profiles of the allochthonous gut microbiota community profile [38]. The increase in the Firmicutes phylum has been verified in different fish species by the dietary inclusion of *H. illucens* meal [57,58,59,60]. To date, no single component has been identified as responsible for these microbiota shifts. Several hypotheses are being postulated, namely insect species [59]; insect microbiota profile [61]; insect development stage [57]; rearing substrate [62,63]; medium-chain fatty acids profiles present in IM (e.g., lauric acid, C12:0) [64]; insect antimicrobial peptides, which can vary between species and life stages [65]; and chitin [57,58,59,66]. Nonetheless, the increase in Firmicutes upon including HM in fish diets is hypothesized to be attended by an increase in chitinolytic bacteria, which would use chitin as a substrate to grow [57,67,68].

The emergence of chitinolytic bacteria is particularly important when feeding IM to fish, as it will promote chitin digestion. Due to its indigestibility in some economically valuable fish species, chitin is often pointed out as the main obstacle to the effective use of IM in diets [1]. However, the Firmicutes increase previously verified in fish fed the HM25 diet [38] was not concomitant with an increase in cultivable aerobic spore-forming chitinolytic bacteria, as verified in this work. On the other hand, diet HEM25, which presents a similar chitin content to diet HM25 (1.8% vs. 1.7%), led to the majority of the chitinolytic FIs obtained. A potential explanation for the emergence of chitinolytic bacteria in fish fed with the HEM25 diet might be the result of the molting processes that lead to exuviae detachment. Secretion of chitinase and peptidase-rich molting fluids during apolysis can lead to higher amounts of N-acetylglucosamine monomers and dimers in HEM25 [69,70,71], which can prompt the proliferation of known chitinolytic families of the Firmicutes phylum, such as *Bacillaceae* and *Planococcaceae* [72,73]. Moreover, during ecdysis, the loosening and splitting of the insect’s old cuticle leads to the disruption of the chitin matrix and, thus, increases the access of enzymes and/or bacteria to this polymer, potentially facilitating its colonization and/or use [21,70,74]. Independently of the underlining mechanisms of microbiota modulation, the increase in cultivable aerobic spore-forming chitinolytic bacteria, coupled with the increased chitinolytic genomic potential previously demonstrated [38], establishes the potential of HEM to modulate the fish gut microbiota toward chitinolytic microorganisms. 

*Bacillus*, the most predominant genus among our FIs (85.2%), has been extensively explored for probiotic supplementation in aquaculture [75,76], with several species being listed in the EFSA list of Qualified Presumption of Safety (QPS) [77]. On the other hand, *Paenibacillus* application in aquaculture is still limited. Both genera share characteristics that make them good probiotic candidates for feed inclusion as they can produce antimicrobial compounds capable of disrupting pathogen growth and/or communication [75,78,79,80,81], can enhance fish growth performance and immune status [76,82,83,84,85,86], and can decompose non-starch polysaccharides (NSPs) and chitin [35,80,87,88].

Within the European Union, potential probiotics must comply with the guidelines of the EFSA Panel on Additives Products or Substances used in Animal Feed before approval for inclusion in aquafeeds [47]. Safety-associated criteria must be fulfilled, including the absence of toxigenic activity. While in *Paenibacillus*, information regarding toxicity and endotoxins production is scarce [80], in *Bacillus*, a comparative pathogenomics analysis using the Virulence Factor Database (VDFB) [89] demonstrated that its endotoxin production might lead to hemolysis. Thus, the determination of hemolytic activity allowed the exclusion of all potentially harmful species such as *B. cereus* and *B. paranthracis*, reducing the putative probiotic candidates to 20 FIs. The absence of acquired antibiotic resistance is another safety-associated criterion required by EFSA for probiotic approval. Using EFSA-established epidemiological cutoff (ECOFF) values [47], antibiotic resistance was found in 19 of the 20 FIs, with only FI832 being susceptible to all tested antibiotics. Bacteria belonging to the *Paenibacillus* genus, for which no genus-specific ECOFF values are available, were readily excluded from further testing, as members of this genus were recently indicated as potential disseminators of antibiotic resistance [51]. On the other hand, the genus *Bacillus* has several species with QPS status (e.g., *B. subtilis, B. licheniformis*), as well as established ECOFF values defined by EFSA [90]. These ECOFF values are, however, defined at the genus level and not species-specific, thus providing limited information for distinguishing between harmful strains with acquired resistance and strains with intrinsic resistances [91,92]. Recently, Agersø et al. [93] determined that erythromycin and chloramphenicol resistance in *B. licheniformis* was associated with an ancient resistome with no mobile elements in the vicinity, suggesting novel species-specific tentative ECOFF values for *B. licheniformis* [92]. Re-evaluating our FIs with these novel ECOFF values would classify almost all of them as susceptible to chloramphenicol (of 32 mg L^−1^). Therefore, and assuming that also in our FIs, resistance might be intrinsic rather than horizontally acquired, we have decided to move on with their characterization for potential application as chitinolytic probiotics. Notwithstanding this assumption, in the future, for EFSA approval as a probiotic, the genetic basis behind the resistance observed will have to be determined for the final selected FI with the most promising chitinolytic activity.

Aiming to maximize chitin digestion in IM and, thus, its bioavailability, FIs were further selected based on their extracellular ability to degrade chitin into N-acetylglucosamine monomers. This selection criterion was chosen as in teleost fish, intestinal uptake of N-acetylglucosamine, or its oligomers, is poorly understood; it is hypothesized that N-acetylglucosamine is the monomeric form that enters the fish enterocytes through diffusion [94]. The ability of bacteria to reduce chitin into its monomers in the extracellular space occurs due to the synergetic action of lytic chitin monooxygenase and endo- and exo-chitinases [95]. These enzymes have been identified in several members of the *Bacillus* genus, such as *B. thuringiensis* [88,96], *B. subtilis* [97,98], and *B. licheniformis* [99,100,101], the closest known species of the four selected isolates (FI645, FI658, FI699, and FI832). ChiA-type chitinases are key players in the chitin degradation process, being the predominant chitinase type in both cultured and uncultured bacteria [102,103,104]. As such, *chiA* has been used as the target gene to correlate the chitinolytic phenotype with chitinase presence [48,105]. In marine environments, primers designed by Ramaiah et al. [48] were shown to be highly effective in amplifying the *chiA* gene of a large array of phylogenetically distant bacteria, including *Bacillus* spp. [48,105]. However, these primers were ineffective for the determination of *chiA* in our four FIs. Confirmation of *chiA* presence was only possible by using primers specifically designed in this work to target the *chiA* gene of *B. licheniformis*. Variations of the *chiA* gene are known to occur in response to the microenvironments occupied by bacteria, potentially altering the protein structure to be better suited to the conditions found [106]. As such, the ineffectiveness of the primers designed by Ramaiah et al. [48] can be the result of a selective pressure resulting from the particular conditions found within the ESB gut, such as temperature and pH. When exposed to ESB gut-like environments regarding temperature and pH, namely 24 °C and pH 7 [44,45], the FIs either increased their activity (FI645, FI658, FI699) or maintained it (FI832) when compared to their activity at 37 °C, differing from other *Bacillus* chitinases which have been shown to decrease their activity at lower temperatures [98,99,107]. Among these, FI645 and FI658 were shown to have the highest chitinolytic activities in ESB gut-like conditions, thus being particularly promising for aquaculture applications. Additionally, increased chitinolytic activity at lower temperatures (close to room temperature) opens novel biotechnological possibilities for these FIs and/or their chitinases as they offer significant advantages, for instance, for the chitin bioconversion industry and fungal control [108,109].

Finally, the four putative FI probiotics should possess essential traits such as high sporulation yields and survivability in the gastrointestinal environment, characteristics that determine their economic viability, industrial scaling, and oral application [110]. At a laboratory scale, all isolates presented high sporulation yields, between 5.25 × 10^8^ cells mL^−1^ and 9.77 × 10^8^ cells mL^−1^, and good sporulation efficiencies, between 46% and 89%, and thus a cost-effective industrial scaling process is anticipated. Moreover, when sequentially exposed to simulated gastric and intestinal conditions, they also presented high survival performances, thus demonstrating the ability to survive passage through the harsh gastrointestinal environment. These are promising results for in vivo application, as the survival observed after gastrointestinal environment simulations indicate that the four FIs will be able to potentially maintain their viability and desirable characteristics which are critical for germination, adhesion, and colonization within the fish intestine, where they will exert their desired probiotic abilities [111,112], namely chitin degradation.

## 5. Conclusions

The data gathered in the present study demonstrate that *H. illucens*-derived meals exert a selective pressure on fish gut microbiota towards an increase in bacteria belonging to the Firmicutes phylum, being in line with our previous findings and the literature. In particular, *H. illucens* exuviae, a novel ingredient, greatly impacted the digesta-associated fish gut microbiota, increasing the number of chitin-metabolizing bacteria. Given the results, it is possible to conclude that *H. illucens* exuviae are the best-suited ingredient to promote the emergence of spore-forming chitinolytic bacteria. This opens encouraging prospects for the use of an unexplored by-product of insect production as a functional ingredient. In the future, to better understand the HEM-induced microbiota modulation, a full characterization of this novel ingredient should be carried out. The application of a series of consecutive selection criteria to the initial pool of aerobic chitinolytic spore-forming bacteria obtained in this study allowed the identification of 4 isolates (FI645, FI658, FI699, and FI832) out of 366 as the most promising to increase chitin digestibility. Among these, FI645 and FI658 produced the best results regarding chitinolytic activity in gut-like conditions, sporulation yield and efficiency, and spore survival after exposure to gut-like conditions. Given the in vitro potential demonstrated by these isolates, in future works they will be evaluated in vivo, in IM-containing diets, to confirm their probiotic potential. The successful selection of these probiotics provides a novel biotechnological approach to optimize chitin digestibility in IM, thus increasing its potential asFM substitute.

## Figures and Tables

**Table 1:** Characterization and identification of the 27 isolates displaying chitinolytic activity.

Isolates ^a^	Diet ^b^	Spores ^c^	Catalase ^d^	Hemolysis ^e^	AbR ^f^	16S rRNA Sequence Analysis	
						Closest Known Species ^g^	% ID
**FI540**	HEM25	+	+	γ	EC	*Bacillus licheniformis*	91.4
FI542	HEM25	+	+	γ	EC	*Bacillus licheniformis*	93.7
FI546	HEM25	+	+	γ	EC	*Bacillus licheniformis*	91.2
FI564	HEM25	+	+	γ	EC	*Bacillus licheniformis*	94.7
**FI568**	HEM25	+	+	β	n/d	*Bacillus tropicus*	100.0
FI590	HM25	+	+	γ	C	*Bacillus licheniformis*	94.5
FI620	CHT5	+	+	γ	KS	*Paenibacillus xylanilyticus*	98.8
FI622	CTR	+	+	γ	SC	*Paenibacillus tundrae*	99.3
**FI633**	HEM25	+	+	β	n/d	*Bacillus cereus*	98.3
**FI635**	HEM25	+	+	β	n/d	*Bacillus cereus*	99.9
**FI640**	HEM25	+	+	β	n/d	*Bacillus paranthracis*	99.9
FI645	HEM25	+	+	γ	EC	*Bacillus licheniformis*	99.7
FI650	HEM25	+	+	γ	EC	*Bacillus* spp.	91.5
**FI652**	HEM25	+	+	β	n/d	*Bacillus clausii*	99.5
FI657	HEM25	+	+	γ	EC	*Bacillus licheniformis*	99.1
FI658	HEM25	+	+	γ	EC	*Bacillus licheniformis*	98.5
FI662	HEM25	+	+	γ	EC	*Bacillus licheniformis*	90.0
FI669	HEM25	+	+	γ	C	*Bacillus licheniformis*	92.1
**FI670**	HEM25	+	+	β	n/d	*Bacillus cereus*	99.0
FI677	HEM25	+	+	γ	EC	*Bacillus licheniformis*	93.3
FI698	CHT5	+	+	γ	KTV	*Paenibacillus dendritiformis*	92.3
FI699	CHT5	+	+	γ	EC	*Bacillus licheniformis*	98.0
FI710	HM25	+	+	γ	EKSC	*Paenibacillus lautus*	99.4
FI760	HEM25	+	+	γ	C	*Bacillus* spp.	92.3
FI779	HEM25	+	+	γ	EC	*Bacillus* spp.	94.4
**FI805**	HEM25	+	+	β	n/d	*Bacillus paranthracis*	99.9
FI832	HM25	+	+	γ	-	*Bacillus licheniformis*	99.7

^a^ Isolates showing strong hemolytic activity are displayed in bold. ^b^ CTR, control fishmeal-based diet; HM, *Hermetia illucens* larvae meal-based diet; HEM, *Hermetia illucens* exuviae meal-based diet; CHT5, control fishmeal-based diet with 5% chitin supplementation. ^c^ Spores detected by phase-contrast microscopy of 24 h cultures in Difco Sporulation Medium (DSM) agar. ^d^ Catalase activity tested by resuspending a colony in a 3% solution of hydrogen peroxide (Sigma). ^e^ Hemolysis determined on Columbia 5% sheep blood agar plates after incubation at 37 °C for 72 h. β-hemolysis, the bacterial hemolytic enzymes completely break down the blood cells; α-hemolysis, the bacterial hemolytic enzymes only partially break down the blood cells; γ-hemolysis corresponds to essentially no hemolytic activity detected. ^f^ Antimicrobial resistance determined by the E-test method against several antibiotics, namely erythromycin (E), kanamycin (K), tetracycline (T), vancomycin (V), streptomycin (S), gentamycin (G), chloramphenicol (C), no resistances (-), not determined (n/d). Resistances to a specific antibiotic are illustrated by the corresponding letters. Resistance was defined according to the EFSA’s breakpoints (EFSA, 2012) for *Bacillus* sp. or other Gram+ bacteria in accordance with each isolate identification. ^g^ Closest known species found using BLASTn of the 16S rRNA gene against a database of non-redundant bacterial sequences from NCBI.

## Data Availability

Not applicable.

## Supplementary Materials

The following supporting information can be downloaded at: https://www.mdpi.com/article/10.3390/biology11070964/s1, Supplementary Figure S1. Alignment of chiA sequences from *B. licheniformis* strains identified with the accession numbers GQ899144, FJ465148, KJ017976, CP042252, CP041154, CP023729; Supplementary Table S1. Ingredient composition and experimental diet proximate analysis (Rangel et al., 2022); Supplementary Table S2. chiA-specific oligonucleotide primer-pairs used in this study; Supplementary Table S3. Antibiotic resistance of the tested strains.
