# Supplementary material for: Isolation of Chitinolytic Bacteria from European Sea Bass Gut Microbiota Fed Diets with Distinct Insect Meals
Source: Biology (Basel). 2022 Jun 25;11(7):964. doi: 10.3390/biology11070964 (PMC9312007; doi:10.3390/biology11070964)
Supplement: Supplementary file 1 [file biology-11-00964-s001.zip › biology-1779410-supplementary.pdf]

Supplementary Material

Isolation of chitinolytic bacteria from European sea bass gut microbiota fed diets with distinct insect meals

F. Rangel[1,2], R. A. Santos[1,2], M. Monteiro[1,2], A. S. Lavrador[1,2], L. Gasco[3], F. Gai[4], A. Oliva-Teles[1,2], P. Enes[1,2], C. R. Serra[1,2]

[1]Department of Biology, Faculty of Sciences, University of Porto, Rua do Campo Alegre, Ed. FC4, 4169-007 Porto, Portugal.
[2]CIMAR/CIIMAR Interdisciplinary Centre of Marine and Environmental Research, University of Porto, Terminal de Cruzeiros do Porto de Leixões, Av. General Norton de Matos s/n, 4450-208 Matosinhos, Portugal.
[3]Department of Agricultural, Forest and Food Sciences, University of Turin, Largo P. Braccini 2, 10095 Grugliasco, TO, Torino, Italy.
[4]Institute of Science of Food Production, National Research Council, Largo P. Braccini 2, 10095 Grugliasco, TO, Torino, Italy.

Correspondence: Paula Enes (enes.ciimar@gmail.com) and Cláudia Serra (claudia.serra@fc.up.pt)

```
                ....|....| ....|....| ....|....| ....|....| ....|....| ....|....| ....|....| ....|....| ....|....| ....|....|
                       155        165        175        185        195        205        215        225        235        245

GQ899144.1      CATGGGGTGC TTATGGAAGG GATTTTCAAG TTTGGGATAT GGACGTTTCG AAAGTCAGCC ACATTAATTA TGCCTTTGCT GATATTTGCT GGGAGGGAAG
FJ465148.1      CATGGGGTGC TTATGGAAGG GATTTTCAAG TTTGGGATAT GGACGTTTCG AAAGTCAGCC ACATTAATTA TGCCTTTGCT GATATTTGCT GGGAGGGAAG
KJ017976.1      CATGGGGTGC TTATGGAAGG GATTTTCAAG TTTGGGATAT GGACGTTTCG AAAGTCAGCC ACATTAATTA TGCCTTTGCT TATATTTGCT GGGAGGGAAG
CP042252.1      CATGGGGTGC TTATGGAAGG GATTTTCAAG TTTGGGATAT GGACGTTTCG AAAGTCAGCC ACATTAATTA TGCCTTTGCT GATATTTGCT GGGAGGGAAG
CP041154.1      CATGGGGTGC TTATGGAAGG GATTTTCAAG TTTGGGATAT GGACGTTTCG AAAGTCAGCC ACATTAATTA TGCCTTTGCT GATATTTGCT GGGAGGGAAG
CP023729.1      CATGGGGTGC TTATGGAAGG GATTTTCAAG TTTGGGATAT GGACGTTTCG AAAGTCAGCC ACATTAATTA TGCCTTTGCT GATATTTGCT GGGAGGGAAG
                                                              Chit_Blic-FW
                ....|....| ....|....| ....|....| ....|....| ....|....| ....|....| ....|....| ....|....| ....|....| ....|....|
                       255        265        275        285        295        305        315        325        335        345

GQ899144.1      GCATGGAAAC CCTGATCCGA CAGGCCCCAA TCCTCAAACG TGGTCATGCC AGGATGAAAA CGGAGTGATC GACGCGCCAA ATGGAACAAT CGTGATGGGC
FJ465148.1      GCATGGGAAC CCTGATCCGA CAGGCCCCAA TCCTCAAACG TGGTCATGCC AGGATGAAAA CGGAGTGATC GACGCGCCAA ATGGAACAAT CGTGATGGGC
KJ017976.1      GCATGGAAAC CCTGATCCGA CAGGAACCAA TCCTCAAACG TGGTCATGCC AGGATGAAAA CGGAGTGATC GACGCGCCAA ATGGAACAAT CGTGATGGGC
CP042252.1      GCATGGGAAC CCTGATCCGA CAGGCCCCAA TCCTCAAACG TGGTCATGCC AGGATGAAAA CGGAGTGATC GACGCGCCAA ATGGAACAAT CGTGATGGGC
CP041154.1      GCATGGGAAC CCTGATCCGA CAGGCCCCAA TCCTCAAACG TGGTCATGCC AGGATGAAAA CGGAGTGATC GACGCGCCAA ATGGAACAAT CGTGATGGGC
CP023729.1      GCATGGGAAC CCTGATCCGA CAGGCCCCAA TCCTCAAACG TGGTCATGCC AGGATGAAAA CGGAGTGATC GACGCGCCAA ATGGAACAAT CGTGATGGGC

                ....|....| ....|....| ....|....| ....|....| ....|....| ....|....| ....|....| ....|....| ....|....| ....|....|
                       355        365        375        385        395        405        415        425        435        445

GQ899144.1      GATCCCTGGA TTGACGCACA AAAGTCAAAT CCCGGGGATG TCTGGGATGA ACCGATCCGC GGCAACTTTA AACAATTGTT GAAGCTGAAA AAGAGCCACC
FJ465148.1      GATCCCTGGA TTGACGCACA AAAGGCAAAT CCCGGGGATG TCTGGGATGA ACCGATCCGC GGCAACTTTA AACAATTGTT GAAGCTGAAA AAGAGCCACC
KJ017976.1      GATCCCTGGA TTGACGCACA AAAGTCAAAT CCCGGGGATG TCTGGGATGA ACCGATCCGC GGCAACTTTA AACAATTGTT GAAGCTGAAA AAGAGCCACC
CP042252.1      GATCCCTGGA TTGACGCACA AAAGGCAAAT CCCGGGGATG TCTGGGATGA ACCGATCCGC GGCAACTTTA AACAATTGTT GAAGCTGAAA AAGAGCCACC
CP041154.1      GATCCCTGGA TTGACGCACA AAAGGCAAAT CCCGGGGATG TCTGGGATGA ACCGATCCGC GGCAACTTTA AACAATTGTT GAAGCTGAAA AAGAGCCACC
CP023729.1      GATCCCTGGA TTGACGCACA AAAGGCAAAT CCCGGGGATG TCTGGGATGA ACCGATCCGC GGCAACTTTA AACAATTGTT GAAGCTGAAA AAGAGCCACC

                ....|....| ....|....| ....|....| ....|....| ....|....| ....|....| ....|....| ....|....| ....|....| ....|....|
                       455        465        475        485        495        505        515        525        535        545

GQ899144.1      CTCATTTGAA AACGTTCATA TCGGTCGGGG GGTGGACTTG GTCTAACCGC TTTTCAGATG TCGCGGCAGA TCCTGCGGCA AGGGAGAATT TCGCCGCTTC
FJ465148.1      CTCATTTGAA AACGTTCATA TCGGTCGGGG GGTGGACTTG GTCTAACCGC TTTTCAGATG TCGCGGCAGA TCCTGCGGCA AGGGAGAATT TCGCCGCTTC
KJ017976.1      CTCATTTGAA AACGTTCATA TCGGTCGGGG GGTGGACTTG GTCTAACCGC TTTTCAGATG TCGCGGCAGA TCCTGCGGCA AGGGAGAATT TCGCCGCTTC
CP042252.1      CTCATTTGAA AACGTTCATA TCGGTCGGGG GGTGGACTTG GTCTAACCGC TTTTCAGATG TCGCGGCAGA TCCTGCGGCA AGGGAGAATT TCGCCGCTTC
CP041154.1      CTCATTTGAA AACGTTCATA TCGGTCGGGG GGTGGACTTG GTCTAACCGC TTTTCAGATG TCGCGGCAGA TCCTGCGGCA AGGGAGAATT TCGCCGCTTC
CP023729.1      CTCATTTGAA AACGTTCATA TCGGTCGGGG GGTGGACTTG GTCTAACCGC TTTTCAGATG TCGCGGCAGA TCCTGCGGCA AGGGAGAATT TCGCCGCTTC

                ....|....| ....|....| ....|....| ....|....| ....|....| ....|....| ....|....| ....|....| ....|....| ....|....|
                       555        565        575        585        595        605        615        625        635        645

GQ899144.1      GGCCGTTGAG TTTTTAAGGA AATACGGGTT TGACGGGGTC GATCTTGACT GGGAATATCC GGTCAGCGGA GGATTGCCGG GGAACAGCAC ACGTCCGGAA
FJ465148.1      GGCCGTTGAG TTTTTAAGGA AATACGGGTT TGACGGGGTC GATCTTGACT GGGAATATCC GGTCAGCGGA GGATTGCCGG GGAACAGCAC ACGTCCGGAA
KJ017976.1      GGCCGTTGAG TTTTTAAGGA AATACGGGTT TGACGGGGTC GATCTTGACT GGGAATATCC GGTCAGCGGA GGATTGCCGG GGAACAGCAC ACGTCCGGAA
CP042252.1      GGCCGTTGAG TTTTTAAGGA AATACGGGTT TGACGGGGTC GATCTTGACT GGGAATATCC GGTCAGCGGA GGATTGCCGG GGAACAGCAC ACGTCCGGAA
CP041154.1      GGCCGTTGAG TTTTTAAGGA AATACGGGTT TGACGGGGTC GATCTTGACT GGGAATATCC GGTCAGCGGA GGATTGCCGG GGAACAGCAC ACGTCCGGAA
CP023729.1      GGCCGTTGAG TTTTTAAGGA AATACGGGTT TGACGGGGTC GATCTTGACT GGGAATATCC GGTCAGCGGA GGATTGCCGG GGAACAGCAC ACGTCCGGAA
                                 Chit_Blic-REV
```

Supplementary Figure S1. Alignment of *chiA* sequences from *B. licheniformis* strains identified with the accession numbers GQ899144, FJ465148, KJ017976, CP042252, CP041154, and CP023729. Sequences were retrieved from NCBI database (https://blast.ncbi.nlm.nih.gov) and aligned using ClustalW algorithm with Molecular Evolutionary Genetics Analysis (MEGA) software version 10.2.6 (Kumar et al. 2018). Conserved regions of aligned sequences highlighted in blue were chosen to design oligonucleotide primers Chit_Blic_FW and Chit_Blic_REV design using Primer3 software (Rozen and Skaletsky 2000).

Supplementary tables

Supplementary Table S1. Ingredient composition and experimental diet proximate analysis (Rangel et al., 2022).

	Diets				
	CTR	HM25	HEM25	TM25	CHIT5
Ingredients (% dry weight basis)					
Fish meal[a]	44.0	24.4	21.6	19.7	45.1
Soluble fish protein concentrate[b]	2.0	2.0	2.0	2.0	2.0
Hermetia illucens larvae meal[c]	-	25.0	-	-	-
Hermetia illucens exuviae meal[d]	-	-	25.0	-	-
Tenebrio molitor larvae meal[e]	-	-	-	25.0	-
Corn gluten[f]	5.0	5.0	5.0	5.0	5.0
Soybean meal[g]	7.5	7.5	7.5	7.5	7.5
Wheat meal[h]	27.9	20.4	22.1	25.9	21.9
Fish oil	9.9	11.7	11.7	10.5	9.7
Vitamin premix[i]	1.0	1.0	1.0	1.0	1.0
Mineral premix[j]	1.0	1.0	1.0	1.0	1.0
Choline chloride (50%)	0.5	0.5	0.5	0.5	0.5
Binder[k]	1.0	1.0	1.0	1.0	1.0
Taurine[l]	0.2	0.2	0.2	0.2	0.2
Dibasic calcium phosphate	-	0.3	1.4	0.7	-
Chitin[m]	-	-	-	-	5.0
Proximate analyses (% dry weight basis)					
Dry matter	93.6	92.8	89.7	93.4	93.6
Crude protein	46.0	45.4	45.2	45.3	46.3
Crude fat	17.8	17.9	18.1	17.8	18.2
Ash	7.8	8.3	8.6	6.7	7.7
Chitin	-	1.7	1.8	1.3	5.0

[a]Sorgal, S.A. Ovar, Portugal (CP: 72.4% DM; GL: 17.0% DM).
[b]Sorgal, S.A. Ovar, Portugal (CP: 79.7% DM; GL: 7.14% DM).
[c]Black soldier fly larvae meal (CP: 55.4% DM; GL: 10.9% DM; chitin 6.8% DM).
[d]Black soldier fly exuviae meal (CP: 64.3% DM; GL: 8.1% DM; chitin 7.2% DM).
[e]Yellow mealworm larvae meal (CP: 69.5% DM; GL: 14.1% DM; chitin 5.2% DM).
[f]Sorgal, S.A. Ovar, Portugal (CP: 69.9% DM; GL: 3.3% DM).
[g]Sorgal, S.A. Ovar, Portugal (CP: 54.2% DM; GL: 1.8% DM).
[h]Sorgal, S.A. Ovar, Portugal (CP: 13.8% DM; GL: 1.1% DM).
[i]Vitamins (mg kg^{-1} diet): retinol, 18,000 (IU kg^{-1} diet); cholecalciferol, 2000 (IU kg^{-1} diet); α-tocopherol, 35; menadione sodium bisulfate, 10; thiamine, 15; riboflavin, 25; Ca pantothenate, 50; nicotinic acid, 200; pyridoxine, 5; folic acid, 10; cyanocobalamin, 0.02; biotin, 1.5; ascorbyl monophosphate, 50; inositol, 400.
[j]Minerals (mg kg^{-1} diet): cobalt sulfate, 1.91; copper sulfate, 19.6; iron sulfate, 200; sodium fluoride, 2.21; potassium iodide, 0.78; magnesium oxide, 830; manganese oxide, 26; sodium selenite, 0.66; zinc oxide, 37.5; dibasic calcium phosphate, 5.93 (g kg^{-1} diet); potassium chloride, 1.15 (g kg^{-1} diet); sodium chloride, 0.44 (g kg^{-1} diet).
[k]Aquacube. Agil, UK.
[l]Feed-grade taurine, Sorgal, S.A. Ovar, Portugal.
[m]Chitin from shrimp shells, practical grade powder, Sigma-Aldrich.

Supplementary Table S2. *chiA*-specific oligonucleotide primer pairs used in this study.

Primer Name	Primer Sequence (5´- 3´)	Amplicon size	Reference
Chit_sm_FW<br>Chit_sm_REV	GATATCGACTGGGAGTTCCC<br>CATAGAAGTCGTAGGTCATC	225 bp	Ramaiah et al. (2000)
Chit_Blic-FW<br>Chit_Blic-REV	ACGTTTCGAAAGTCAGCCAC<br>CCCGTCAAACCCGTATTTCC	395 bp	This study

Supplementary Table S3. Antibiotic resistance of the tested strains. Two different sets off cut-off values were used. Bacillus were evaluated using the Bacillus sp. cut-off values: erythromycin (E) (4 mg/L), kanamycin (K) (8 mg/L), tetracycline (T) (8 mg/L), vancomycin (V) (4 mg/L), streptomycin (S) (8 mg/L), gentamycin (G) (4 mg/L), chloramphenicol (C) (8 mg/L). Paenibacillus sp. were evaluated with the cut-off values attributed to other Gram+ bacteria: E (1 mg/L), K (16 mg/L), T (2 mg/L), V (4 mg/L), S (8 mg/L), G (4 mg/L), C (4 mg/L).

Isolate	Diet[a]	Closest known species[b]	MIC[c]						
			E	K	T	V	S	G	C
FI540	HEM25	*Bacillus licheniformis*	>256[R]	0.5	0.19	0.75	3	0.094	16[R]
FI542	HEM25	*Bacillus licheniformis*	>256[R]	0.5	0.19	0.75	3	0.094	24[R]
FI546	HEM25	*Bacillus licheniformis*	>256[R]	0.5	0.19	0.5	2	0.094	24[R]
FI564	HEM25	*Bacillus licheniformis*	>256[R]	0.5	0.19	0.5	3	0.094	12[R]
FI590	HM25	*Bacillus licheniformis*	0.25	1	0.5	1	6	0.19	32[R]
FI620	CHT5	*Paenibacillus xylanilyticus*	0.125	16[R]	0.125	1.5	>1024[R]	0.5	2
FI622	CTR	*Paenibacillus tundrae*	1[R]	2	0.094	1	>1024[R]	0.125	16[R]
FI645	HEM25	*Bacillus licheniformis*	>256[R]	4	4	2	6	0.75	48[R]
FI650	HEM25	*Bacillus spp.*	16[R]	0.5	0.5	0.75	2	0.064	16[R]
FI657	HEM25	*Bacillus licheniformis*	>256[R]	0.75	0.5	1	3	0.19	16[R]
FI658	HEM25	*Bacillus licheniformis*	3[H]	1.5	0.75	3	4	1	16[R]
FI662	HEM25	*Bacillus licheniformis*	8[R]	1.5	0.5	0.5	2	0.094	16[R]
FI669	HEM25	*Bacillus licheniformis*	3	0.5	0.38	0.5	3	0.094	16[R]
FI677	HEM25	*Bacillus licheniformis*	>256[R]	0.5	0.5	0.5	2	0.064	16[R]
FI698	CHT5	*Paenibacillus dendritiformis*	0.125	32[R]	12[R]	32[R]	4	1	1.5
FI699	CHT5	*Bacillus licheniformis*	16[R]	1	0.5	2	4	0.19	8[R]
FI710	HM25	*Paenibacillus lautus*	16[R]	64[R]	4[R]	1	384[R]	1	48[R]
FI760	HEM25	*Bacillus spp.*	3	0.5	0.38	0.5	3	0.094	16[R]
FI779	HEM25	*Bacillus spp.*	6[R]	0.38	0.19	0.5	2	0.25	16[R]
FI832	HM25	*Bacillus licheniformis*	0.19	0.05	0.05	1	2	0.19	3

[a]CTR, control fishmeal-based diet; HM, *Hermetia illucens* larvae meal-based diet; HEM, *Hermetia illucens* exuviae meal-based diet; CHT5, control fishmeal-based diet with 5% chitin supplementation. [b]Closest known species found using BLASTn of the 16S rRNA gene against a database of non-redundant bacterial sequences from NCBI. [c]MIC, minimum inhibitory concentration. [R]Resistant according to the EFSA's breakpoints (EFSA 2012). [H]Presents heteroresistant colonies.
